# Cardiovascular events and mortality among patients and controls with calcified carotid artery atheromas on panoramic radiographs: a 10-year follow-up of the PAROKRANK study

**DOI:** 10.1093/dmfr/twag016

**Published:** 2026-03-16

**Authors:** Hessamoddin Faghihian, Eva Levring Jäghagen, Jan Ahlqvist, Kåre Buhlin, Anna Norhammar, Ulf Näslund, Nils Gustafsson

**Affiliations:** Oral and Maxillofacial Radiology, Department of Odontology, Umeå University, SE-90187 Umeå, Sweden; Umeå Centre for Gender Studies, Umeå University, SE-90187 Umeå, Sweden; Oral and Maxillofacial Radiology, Department of Odontology, Umeå University, SE-90187 Umeå, Sweden; Oral and Maxillofacial Radiology, Department of Odontology, Umeå University, SE-90187 Umeå, Sweden; Division of Periodontology, Institute of Odontology, Karolinska Institute, SE-14104 Huddinge, Sweden; Department of Oral and Maxillofacial Diseases, P.O. Box 3, 00014 University of Helsinki, Helsinki, Finland; Cardiology, Department of Medicine, Karolinska Institute, SE-17177 Stockholm, Sweden; Department of Physiology, Capio Siant Göran’s Hospital, SE-11219 Stockholm, Sweden; Cardiology, Department of Public Health and Clinical Medicine, Umeå University, SE-90187 Umeå, Sweden; Oral and Maxillofacial Radiology, Department of Odontology, Umeå University, SE-90187 Umeå, Sweden

**Keywords:** atherosclerosis, carotid arteries, cardiovascular disease, panoramic radiography

## Abstract

**Objectives:**

This study investigated the long-term risk of cardiovascular events and all-cause mortality among participants with calcified carotid artery atheroma (CCAA) on panoramic radiographs (PRs) in the Periodontitis and its Relation to Coronary Artery Disease (PAROKRANK) study.

**Methods:**

In the multicenter, multidisciplinary PAROKRANK study, 805 patients with a first myocardial infarction (MI) and 805 matched controls without MI were recruited at 17 hospitals in Sweden. At baseline, the participants were examined with PR, and in 737 patients and 739 controls, the carotid artery region was assessable. Calcified carotid artery atheromas were identified in 251 (34%) patients and 205 (28%) controls at baseline. The primary endpoint was defined as the first occurrence of all-cause mortality, non-fatal MI, non-fatal stroke, or hospitalization following heart failure after a mean follow-up of 10 years. The risks of cardiovascular events and death were evaluated using event survival analysis and regression models.

**Results:**

Participants with bilateral CCAAs, regardless of whether they were patients or controls, had significantly higher mortality and morbidity than those without CCAA (*P *< .001). The risk of cardiovascular events was increased in the presence of bilateral CCAAs among both controls (hazard ratio = 1.96 [95% CI, 1.21–3.16], *P *= .006) and patients (1.67 [1.15–2.43], *P *= .007).

**Conclusion:**

Bilateral CCAAs on PRs were an indicator of an increased risk of future cardiovascular events and early death among both controls and patients in the PAROKRANK study. Therefore, dentists can detect CCAA on PR and contribute to identifying individuals in need of medical attention and treatment of cardiovascular disease to prevent morbidity and early death.

## Introduction

Cardiovascular disease (CVD) is a major contributor to global mortality and morbidity and, according to the latest version of Global Burden of Disease, its prevalence has increased since 1990.^1^^,^^2^ The main underlying pathophysiological mechanism for CVD is atherosclerosis, which is characterized by the accumulation of lipids, inflammatory cells, and fibrous tissue within the arterial wall, leading to the formation of atherosclerotic plaques. Over time, plaques may become calcified, leading to arterial stiffening and detection on radiographic examinations.^3^

Carotid atherosclerotic disease is a manifestation of systemic atherosclerosis and reflects an underlying generalized CVD process. It is estimated to account for 15%-20% of strokes.^4^ Recent global estimates indicate that carotid plaque affects approximately 21% of adults aged 30-79 years.^5^ The prevalence increases markedly with age, exceeding 60% in those aged ≥60 years according to the population-based VIPVIZA cohort study.^6^

**Figure 1:**
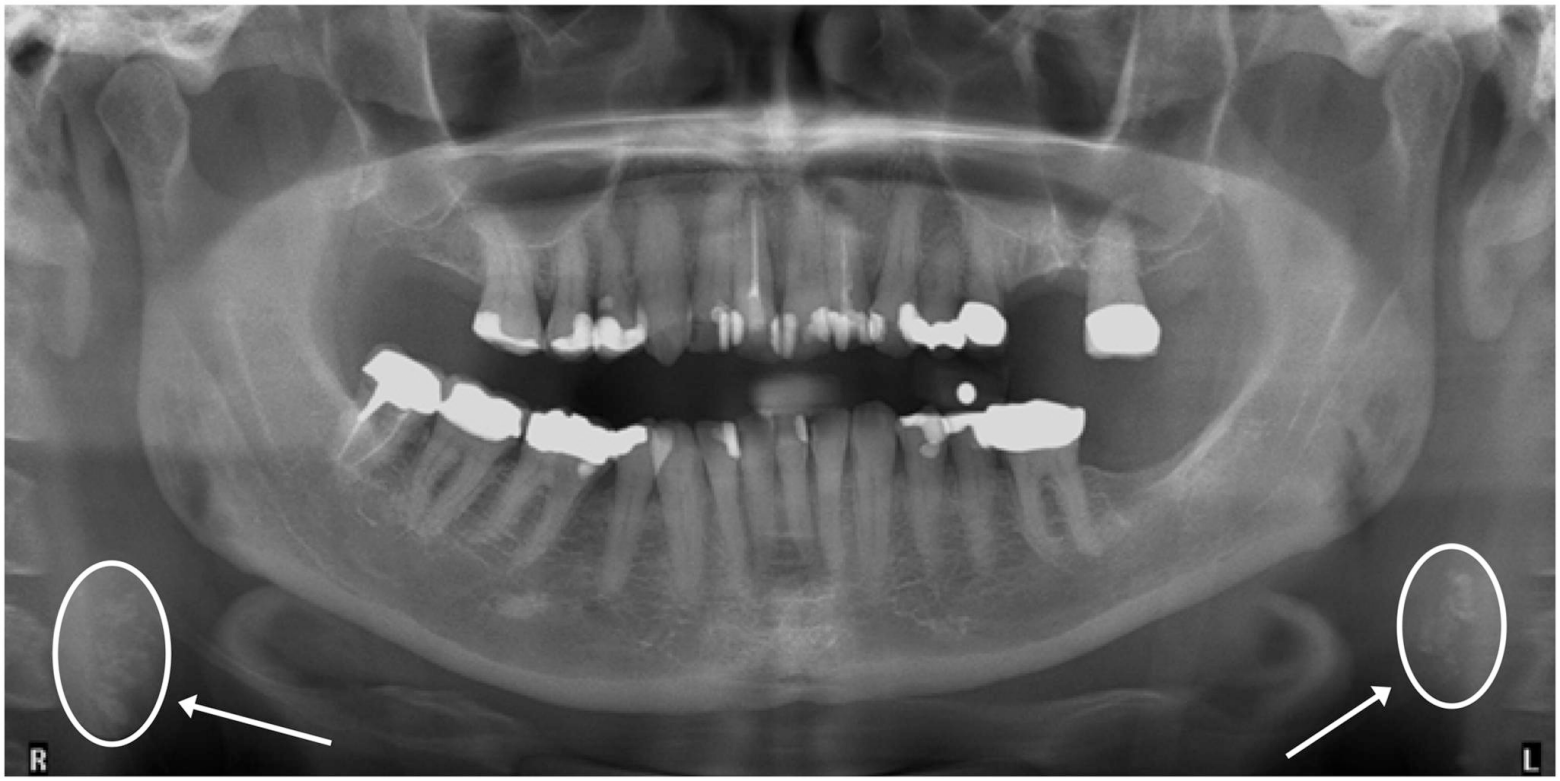
Bilateral calcified carotid artery atheroma (enhanced and marked with white rings) depicted on a panoramic radiograph.

The detection of asymptomatic subclinical carotid plaques can be important because it indicates an increased risk of cardiovascular events, such as stroke and myocardial infarction (MI).^7^ Early detection can contribute to early preventive treatment, but a consensus has not yet been reached on effective preventive measures.^8^ In current clinical practice, asymptomatic subclinical plaque is detected by ultrasound (US) examination or computed tomography (CT), but this requires that the patients actively seek medical care. However, early signs of carotid artery disease can also be observed through incidental findings of calcifications in the area of the carotid arteries on panoramic radiographs (PRs), referred to as calcified carotid artery atheromas (CCAAs).^9–11^ These calcifications typically appear adjacent to the C3-C4 vertebra (Figure 1).

Previous studies have shown that CCAAs on PRs can provide direct evidence of vascular pathology.^12^^,^^13^ When compared to other imaging modalities, such as color Doppler US and CT, the identification of CCAAs on PRs has yielded moderate to high sensitivity and specificity, supporting the potential to detect asymptomatic patients.^14^^,^^15^ CCAAs are seen on PRs in 84% of patients with carotid stenosis ≥50%.^16^ The detection of CCAAs on PRs also strongly correlates with carotid atherosclerosis determined by carotid US.^17^ Furthermore, within the Swedish Periodontitis and its Relation to Coronary Artery Disease (PAROKRANK) cohort,^18^ patients with a recent history of MI have been reported to have a significantly higher prevalence of CCAAs on PRs compared with controls without MI at baseline.^11^ Therefore, assessing CCAAs on PRs can provide an opportunistic approach to identifying people at risk of cerebrovascular and cardiovascular events.

The present study was based on the large multicenter PAROKRANK cohort and aimed to evaluate the association between CCAAs detected on PRs and all-cause mortality, as well as cardiovascular and cerebrovascular outcomes after a mean follow-up of 10 years. This study also explored the potential prognostic value of detecting CCAAs on PRs.

## Method

### Ethical approval

The PAROKRANK study, including the follow-up period, received ethical approval from the Regional Ethics Committee in Stockholm (Dnr: 2008/152-31/2; 2017/1803-32; 2018/2210-32; 2019/02871; 2023/03203-02). Written informed consent was obtained from all participants, and the study was conducted in accordance with the Declaration of Helsinki.

### Population

PAROKRANK is a multicenter, case-control study including 1610 participants recruited from 17 hospitals in Sweden between 2010 and 2014 (mean age ± SD, 62 ± 8 years). The study population consisted of 805 patients (151 women) diagnosed with a recent first acute MI and 805 controls (151 women) without a history of MI, randomly selected from the population register and matched to the patients by age, sex, and postal code/residence address. The inclusion criteria for patients were age <75 years after hospitalization with a first MI according to international diagnostic criteria. Individuals with prior MI, heart valve surgery, or other major conditions precluding study participation were excluded.

Panoramic radiography was performed in 797 patients and 796 controls; the remaining 23 participants were not examined for logistical reasons. However, the PRs of 59 patients and 52 controls were excluded due to overexposure, compression artifacts, or the carotid artery region not being depicted. A more detailed breakdown of the exclusions is described in Gustafsson et al.^11^ A total of 737 patients and 739 controls were included in this study.

### Radiological assessment of CCAA

The PRs were assessed according to Gustafsson et al.^11^ Briefly, PR images were assessed under standardized conditions by 2 specialists in oral and maxillofacial radiology who were blinded to all participant data. For each side (left and right), CCAA was recorded as either present or absent. Distinctions were carefully made between CCAA and other calcifications in the area, such as the hyoid bone, calcified thyroid, and triticeous cartilage. Any disagreements on findings were resolved by consensus. The inter- and intra-observer agreement was high (κ = 0.78 and 0.80-0.85, respectively).

### Follow-up and endpoints

The unique personal identity number assigned to all Swedish residents enabled complete follow-ups for all individuals included in this study. Baseline in patients comprised the dental examination performed 6-10 weeks following the acute MI. The primary endpoint was defined as the first occurrence of any of the following events: all-cause mortality, non-fatal MI, non-fatal stroke, or hospitalization for heart failure. A mean 10-year follow-up of all study participants was conducted until the time of death or 31 December 2022. Through record linkage, the date and cause of death were obtained from the Swedish Cause of Death Register, and information on morbidity was obtained from the Swedish National Patient Register according to the International Classification of Diseases (ICD-10) codes.

### Statistical analysis

Descriptive statistics were used to summarize baseline demographic, clinical, and laboratory variables for both patients and controls. Independent *t*-tests and chi-squared tests were used to draw between-group comparisons for continuous and categorical variables, respectively.

Kaplan-Meier survival analyses were performed separately for patients and controls to estimate time-to-event distributions across the 3 groups defined by CCAA status on PRs: no calcification, unilateral CCAA, and bilateral CCAAs. Survival time was defined as the time from dental examination to the occurrence of death or any major cardiovascular event, such as MI, stroke, or hospitalization due to heart failure; individuals without events were censored at a mean of 10 years after inclusion. Survival curves for each group were compared using a global log-rank test to assess overall differences in survival distributions, followed by pairwise log-rank tests.

A Cox proportional hazards regression model was created to assess whether CCAAs detected on PRs are predictive of future cardiovascular events. CCAA status on PR, classified as absent (no CCAA), unilateral, or bilateral, served as the main variable in the model. The model was adjusted for key cardiovascular covariates, including sex, study group (patient or control), body mass index, systolic blood pressure, smoking status, and the use of lipid-lowering or anticoagulant drugs. Violations of the proportional hazard assumption were controlled for via Schoenfeld residuals and addressed through stratification and adding a log-transformed time interaction. Hazard ratios (HRs) and 95% CIs were estimated relative to the reference category of no CCAA.

Fine-Gray subdistribution hazard models were applied to further explore the relationship between CCAAs and specific cardiovascular outcomes while accounting for competing risks. The outcome variable represented 4 mutually exclusive event types: death, non-fatal MI, hospitalization due to heart failure, and non-fatal stroke. Separate Fine-Gray models were fitted for each event type, and subdistribution HRs (sHRs) with 95% CIs were reported for unilateral and bilateral CCAAs compared to no CCAA. Cumulative incidence functions were derived from the Fine-Gray subdistribution hazard models to estimate the probability of each cardiovascular outcome over time while accounting for competing risks.

*P *< .05 was considered significant. In the case of multiple testing, the Bonferroni correction was used. All analyses were conducted in R (R Foundation for Statistical Computing, Vienna, Austria, v. 4.5).

## Results

As previously reported by Gustafsson et al,^11^ baseline data were consistent with earlier findings (Table 1). The patients had a significantly higher prevalence of family history of CVD (*P *< .001), current smoking (*P *< .001), and newly detected diabetes (*P *= .01) than controls. CCAAs were also more frequently detected on PRs in patients (34.1%) than in controls (27.7%, *P *= .009), with bilateral CCAAs having a notably higher prevalence in patients (14.1% vs. 9.2%, *P *= .003; odds ratio [OR] = 1.62, 95% CI: 1.17–2.24). When stratified by sex, CCAAs were present in 33.0% of men in the patient group vs. 26.7% of men in the control group (*P *= .017; OR = 1.24, 95% CI: 1.04–1.47) and in 38.4% of women in the patient group vs. 31.9% of women in the control group (*P *= .256; OR = 0.753, 95% CI: 0.461–1.229). Bilateral CCAAs also had a higher prevalence in patients among both men (12.5% vs. 8.7%, *P *= .006; OR = 1.53, 95% CI: 1.04–2.26) and women (21.0% vs. 11.1%, *P *= .047; OR = 1.99, 95% CI: 1.01–3.92).

**Table 1:** Baseline characteristics of patients and controls with between-group comparisons.

Variables		Patients (*n *= 737)	Controls (*n *= 739)	*P*-value
Age (years)		61.9 ± 7.8	62.3 ± 7.8	–
Number of women		138 (18.7%)	144 (19.5%)	–
Known family history of cardiovascular disease		276 (43.3%)	166 (26.0%)	**<.001**
Medical history				
	Hypertension	266 (36.2%)	252 (34.2%)	.42
	Peripheral artery disease	19 (2.6%)	10 (1.4%)	.90
	Stroke	20 (2.7%)	16 (2.2%)	.49
	Diabetes mellitus	75 (10.2%)	59 (8%)	.13
	Rheumatic disease	150 (20.7%)	126 (17.2%)	.08
	Pulmonary disease	96 (13.3%)	77 (10.5%)	.09
	Kidney disease	31 (4.2%)	30 (4.1%)	.88
	Cancer (not oral)	52 (7.1%)	45 (6.1%)	.45
	Depression	70 (9.5%)	63 (8.5%)	.51
Smoking habits				
	Current	95 (12.9%)	127 (17.2%)	**<.001**
	Previous	422 (57.3%)	319 (43.2%)	
	Never	219 (29.7%)	292 (39.6%)	
Anthropometric measurements				
	Waist circumference (cm)	99.3 ± 11.2	98.2 ± 11.7	.34
	Body mass index (kg m^−^²)	27.1 ± 3.9	26.9 ± 4.1	.26
Blood pressure (mm Hg)				
	Systolic blood pressure	129.5 ± 16.9	137.4 ± 17.3	.17
	Diastolic blood pressure	76.6 ± 10.0	83.5 ± 10.2	.78
Blood tests				
	Cholesterol (mmol L^−1^)	3.9 ± 0.9	5.6 ± 1.1	**<.001**
	Triglycerides (mmol L^−1^)	1.3 ± 0.9	1.5 ± 1.3	**.009**
	Fibrinogen (g L^−1^)	3.4 ± 0.8	3.2 ± 0.7	**.02**
	High-sensitivity CRP (mg L^−1^)	2.2 ± 2.4	2.2 ± 2.5	.69
	White blood cell count (×10⁹ L^−1^)	6.6 ± 4.4	5.6 ± 2.1	**.01**
	Platelet count (×10⁹ L^−1^)	238.8 ± 63.3	233.5 ± 59.3	.20
	HbA1c (mmol/mol)	40.8 ± 8.2	39.1 ± 7.9	.13
Glycemic state (OGTT)				
	Fasting plasma glucose (mmol L^−1^)	6.0 ± 1.5	5.6 ± 1.2	**.08**
	120-min post-load plasma glucose (mmol L^−1^)	7.0 ± 2.5	6.3 ± 2.4	.16
Diabetes and impaired glucose tolerance (IGT)				
	Newly detected diabetes	66 (9.0%)	41 (5.5%)	**.01**
	Previously known + newly detected	141 (19.3%)	101 (13.7%)	**.004**
Pharmacological treatment				
	Calcium-channel blockers	638 (86.6%)	92 (12.4%)	**<.001**
	Beta-blockers	619 (84.0%)	102 (13.8%)	**<.001**
	Aspirin (acetylsalicylic acid)	647 (87.8%)	75 (10.1%)	**<.001**
	ACE inhibitors	632 (85.8%)	94 (12.7%)	**<.001**
	Antidepressants	33 (4.5%)	46 (6.2%)	.19
CCAA findings on PRs				
	Absent	486 (65.9%)	534 (72.3%)	**.009**
	Present (unilateral or bilateral)	251 (34.1%)	205 (27.7%)	
	Only unilateral	147 (19.9%)	137 (18.5%)	.49
	Only bilateral	104 (14.1%)	68 (9.2%)	**.003**

Values are presented as mean ± SD or *n* (%). Abbreviations: CRP = C-reactive protein; HbA1c = hemoglobin A1C; OGTT = oral glucose tolerance test; ACE = angiotensin-converting enzyme; IGT = impaired glucose tolerance; CCAA = calcified carotid artery atheroma; PR = panoramic radiograph. Bold values indicate statistical significance (*P*-value <.05).

Biochemical markers, such as cholesterol, triglycerides, fibrinogen, and white blood cell count, were significantly elevated in patients compared to controls. Use of cardiovascular medications (eg, calcium-channel blockers, beta-blockers, aspirin, angiotensin-converting enzyme inhibitors) was substantially more common in the patient group (all *P *< .001).

**Figure 2:**
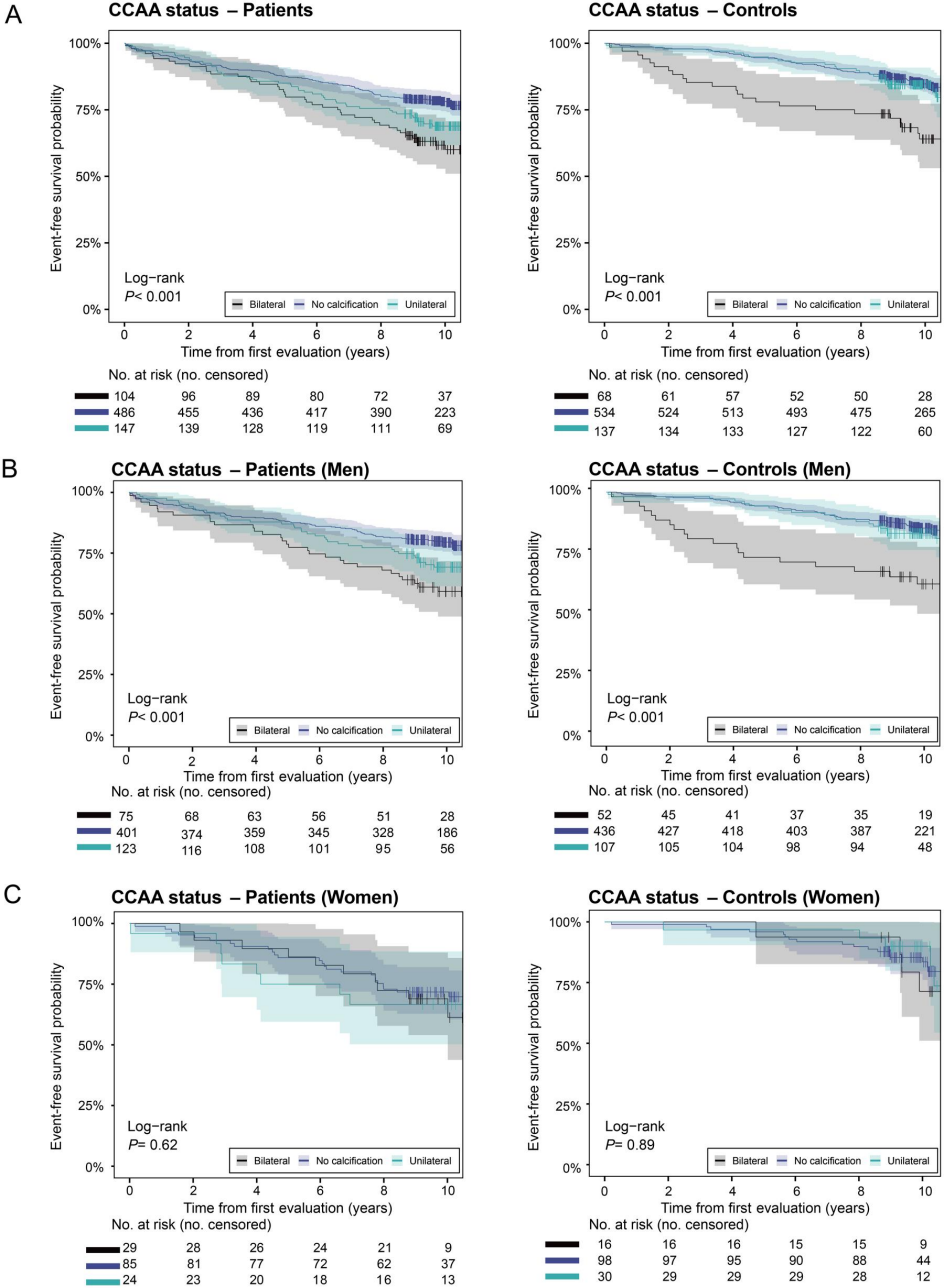
Kaplan-Meier curves of cardiovascular event-free survival by calcified carotid artery atheroma (CCAA) status stratified by study group (A) among men (B) and women (C) participants. Individuals with bilateral CCAAs, both patients and controls, had significantly worse survival based on global log-rank tests (*P *< .001 for patients, *P *< .001 for controls). A significant difference in survival according to CCAA status was also found among men (*P *< .001), but no significant difference was observed among women (*P *= .89 for controls, *P *= .62 for patients).

Kaplan-Meier curves stratified by CCAA status on PR showed significant differences in cardiovascular event-free survival probability among both patients (*P *< .001) and controls (*P *< .001, Figure 2). In patients, bilateral CCAAs were associated with significantly lower survival probability compared to patients without CCAA (adjusted *P *< .001), but there was no significant difference in event survival probability between unilateral and bilateral CCAAs. Controls with bilateral CCAAs had lower survival probability than controls with no calcification (*P *< .001) and controls with unilateral CCAA (*P *= .017). Among both patients and controls, no significant difference was observed between unilateral CCAA and no calcification (*P *= 1.00, Table 2).

**Table 2:** Pairwise comparisons of survival curves based on calcified carotid artery atheroma (CCAA) status.

Study group	Comparison	Adjusted *P*-value^a^
Patients	No calcification vs bilateral CCAA	**<.001**
	No calcification vs unilateral CCAA	.08
	Unilateral CCAA vs bilateral CCAA	.13
Controls	No calcification vs bilateral CCAA	**<.001**
	No calcification vs unilateral CCAA	1.00
	Unilateral CCAA vs bilateral CCAA	**<.05**

a Bonferroni-adjusted log-rank *P*-values. Abbreviation: CCAA = calcified carotid artery atheroma. Bold values indicate statistical significance (*P*-value <.05).

Stratification by sex showed a significant difference in cardiovascular event-free survival according to CCAA status among men (*P *< .001, both patients and controls), but the difference was not significant among women (patients: *P *= .62, controls: *P *= .89). This pattern remained unchanged when patients and controls were merged into 1 group and stratified by sex, with significance for men (*P *< .001) but not for women (*P *= .45, Figure 2).

In the multivariable Cox regression model of the total cohort, bilateral CCAAs were significantly associated with an increased risk of future cardiovascular events and death compared to no CCAA (all participants: HR = 1.76, 95% CI: 1.31–2.36, *P *< .001). However, a significant association was not found for unilateral CCAA (HR = 1.08, 95% CI: 0.82–1.43, *P *= .58). When analyzed separately, bilateral CCAAs remained a significant indicator of increased risk among both patients (HR = 1.67, 95% CI: 1.15–2.43, *P *= .007) and controls (HR = 1.96, 95% CI: 1.21–3.16, *P *= .006). Unilateral CCAA was not significantly associated with events in either subgroup (Table 3).

**Table 3:** Multivariable Cox proportional hazards regression.

Variable		All participants	Patients	Controls
Age^a^		1.05 (1.02–1.07), ***P *< .001**	1.05 (1.03–1.08), ***P *< .001**	1.05 (1.00–1.10), ***P *= .038**
Body mass index (BMI)		1.03 (1.00–1.06), ***P *= .027**	1.02 (0.98–1.05), *P *= .43	1.07 (1.03–1.12), ***P *< .01**
Hypertension		1.18 (0.93–1.50), *P *= .17	1.15 (0.85–1.57), *P *= .37	1.34 (0.92–1.96), *P *= .13
Smoking	No	Ref.	Ref.	Ref.
	Current	0.82 (0.64–1.06), *P *= .13	0.98 (0.69–1.40), *P *= .92	0.69 (0.48–0.99), ***P *= .045**
	Former	1.43 (1.06–1.91), ***P *= .018**	1.71 (1.19–2.45), ***P *= .003**	0.84 (0.46–1.53), *P *= .56
Systolic blood pressure^a^		0.99 (0.98–1.00), ***P *= .026**	0.98 (0.97–0.99), ***P *= .003**	1.01 (1.00–1.02), *P *= .31
High-density lipoprotein (HDL) cholesterol^a^		0.61 (0.37–1.01), *P *= .052	0.67 (0.43–1.04), *P *= .075	0.88 (0.55–1.39), *P *= .58
Statin use		0.81 (0.57–1.16), *P *= .25	0.49 (0.26–0.92), ***P *= .026**	0.68 (0.41–1.15), *P *= .15
Aspirin use		1.56 (1.09–2.23), ***P *= .014**	0.70 (0.33–1.52), *P *= .37	1.12 (0.66–1.90), *P *= .67
CCAA status	Absent	Ref.	Ref.	Ref.
	Unilateral CCAA	1.08 (0.82–1.43), *P *= .58	1.19 (0.83–1.71), *P *= .33	0.94 (0.60–1.49), *P *= .80
	Bilateral CCAA	1.76 (1.31–2.36), ***P *< .001**	1.67 (1.15–2.43), ***P *= .0068**	1.96 (1.21–3.16), ***P *= .0059**

a Variable was modified by interaction with log(t) to avoid violation of proportional hazard assumption.

Values are presented as hazard ratio (95% CI). The regression assessed the association between demographic and clinical data, calcified carotid artery atheroma (CCAA) status on panoramic radiographs, and the risk of cardiovascular events separately in all participants, patients, and controls. Model statistics-all participants: concordance = 0.667 (SE = 0.015), likelihood ratio test *P *< .0001; patients: concordance = 0.645 (SE = 0.021), likelihood ratio test *P *< .0001; controls: concordance = 0.701 (SE = 0.021), likelihood ratio test *P *< .0001. Bold values indicate statistical significance (*P*-value <.05).

The results of Fine-Gray risk regression analysis of all participant (patients and controls), showed that bilateral CCAAs were significantly associated with an increased risk of death (sHR = 2.28, 95% CI: 1.43-3.62, *P *< .001), MI (sHR = 1.76, 95% CI: 1.08-2.88, *P = *.023), and stroke (sHR = 2.48, 95% CI: 1.43–4.30, *P *< .01; Table 4). These associations remained significant among patients, with greater sHRs for death and stroke (2.59 and 2.67, respectively). Among controls, trends were similar but weaker and did not consistently reach significance.

**Table 4:** Subdistribution hazard ratios (sHRs) from Fine–Gray competing risks models assessing the association between calcified carotid artery atheroma (CCAA) status and specific cardiovascular outcomes.

Variable	Outcome	Study group	sHR	95% CI	*P*-value
Unilateral CCAA	Death	All	1.01	0.6–1.71	.96
		Controls	0.89	0.41–1.91	.76
		Patients	1.17	0.57–2.43	.66
	Heart failure	All	2.34	1.1–4.99	**.028**
		Controls	5.92	1.67–20.94	**.005**
		Patients	1.28	0.46–3.59	.64
	Myocardial infarction	All	1.18	0.75–1.87	.48
		Controls	0.71	0.25–2.06	.53
		Patients	1.29	0.77–2.16	.33
	Stroke	All	1.36	0.78–2.38	.28
		Controls	0.95	0.39–2.32	.91
		Patients	1.82	0.87–3.78	.11
Bilateral CCAAs	Death	All	2.28	1.43–3.62	**<.001**
		Controls	1.97	0.95–4.07	.067
		Patients	2.59	1.39–4.82	**.002**
	Heart failure	All	0.7	0.16–3.02	.63
		Controls	1.99	0.22–17.97	.54
		Patients	0.36	0.05–2.71	.32
	Myocardial infarction	All	1.76	1.08–2.88	**.023**
		Controls	2.23	0.89–5.56	.086
		Patients	1.41	0.79–2.5	.24
	Stroke	All	2.48	1.43–4.3	**.001**
		Controls	2.3	0.98–5.4	.056
		Patients	2.67	1.28–5.57	**.009**

Separate models were run for the total cohort, patients with history of MI, and controls. sHRs and 95% CIs account for competing risks. Abbreviations: CCAA = calcified carotid artery atheroma; HF = heart failure; MI = myocardial infarction; sHR = subdistribution hazard ratio. Bold values indicate statistical significance (*P*-value <.05).

**Figure 3:**
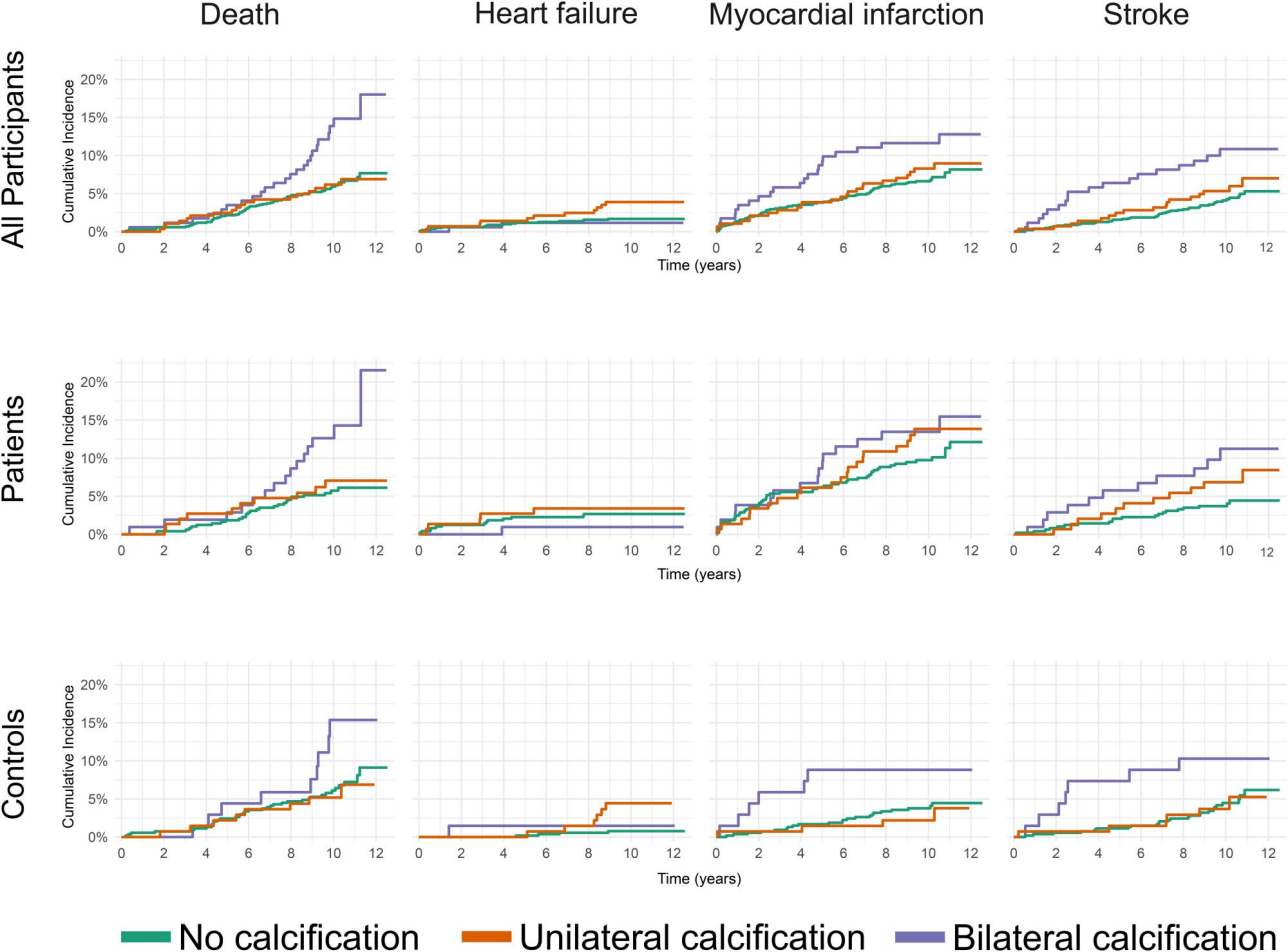
Cumulative incidence functions for specific cardiovascular events and death stratified by calcified carotid artery atheroma status.

Unilateral CCAA was not significantly associated with MI, stroke or death, but it was linked to a higher risk of heart failure in the full cohort (sHR = 2.34, 95% CI: 1.10–4.99, *P *= .028), mainly in controls (sHR = 5.92, *P *= .006). Cumulative incidence plots showing the trends with respect to each CCAA status are provided in Figure 3.

## Discussion

The main finding of this long-term follow-up study based on the PAROKRANK cohort showed that both patients with MI and controls with bilateral CCAAs on PRs have an increased risk of suffering from cardiovascular events and death compared with individuals without CCAA. This finding aligns with recent evidence that bilateral carotid calcifications are independently associated with future vascular events.^19^^,^^20^ As bilateral CCAAs were found in more than 10% of all participants at baseline, our results indicate that these patients require further examination of cardiovascular risk factors and possible preventive treatment, which is in agreement with previous reports.^21^

In the present study, unilateral CCAA on PR was not associated with a significantly increased risk of cardiovascular events or death, though the Kaplan-Meier curves showed a trend toward increased risk among patients with unilateral CCAA compared to participants without CCAA on PR. However, this trend was not significant, which can be explained by the limited sample size. Despite the absence of an increased risk associated with unilateral CCAA, both unilateral and bilateral CCAAs may warrant clinical attention and appropriate management, especially if combined with other risk factors for CVD.

Bilateral CCAAs on PRs were associated with significantly lower cardiovascular event-free survival among men in the cohort, but not among women. The observed sex-specific pattern may be explained by the fact that being a man is a well-established risk factor for CVD^22^ and that women develop CVD at an older age. In the present cohort, one of the inclusion criteria was age <75 years. Consequently, the number of men was higher at baseline, which contributed to a higher number of events and power in the survival analysis. The non-significant result among women could mean that the prognostic impact of bilateral CCAAs may differ by sex or is less pronounced among women. However, given the lower prevalence of CCAA in women in this study, the analyses may have had limited statistical power to detect associations with future events. Future studies including a larger cohort without an upper age limit are needed to clarify the sex-specific prognostic value of bilateral CCAAs.

In the multivariable Cox regression, bilateral CCAAs nearly doubled the HR of composite cardiovascular events, even after adjusting for age, blood pressure, lipid profile, smoking, and medication use. The Fine-Gray competing-risk analyses reinforced these findings, showing the strongest associations of bilateral CCAAs with stroke and death and a more limited association with MI. The particularly strong association with stroke is expected, as calcifications in the carotid arteries directly reflect a local atherosclerotic burden and risk of embolic cerebrovascular events.^23^^,^^24^ The increased mortality risk likely reflects bilateral calcifications representing a systemic manifestation of atherosclerosis that can extend beyond the carotid arteries.^25–27^

One strength of the present study is that the findings were consistent across both the Cox regression model, which estimates the instantaneous risk of an event at any point in time, and the Fine-Gray competing-risk model, which accounts for the possibility of the occurrence of other outcomes that can preclude the event of interest. This modeling approach reduced bias and improved fitness, whereas the CCAA effects remained stable over time, confirming a persistent link with adverse outcomes.

In addition, the PRs were independently reviewed by 2 specialists in oral and maxillofacial radiology who considered both the presence of CCAA and the need to differentiate it from other calcified structures. The results were based on a consensus of the findings to strengthen the accuracy and reliability of the results. By combining rigorous modeling with expert image evaluation in a large cohort with long-term follow-up, this study provides, to the best of our knowledge, the first long-term evidence that bilateral CCAA detected on PR is associated with an increased risk of future cardiovascular events and early death.

The recently updated guidelines from the European Society for Vascular Surgery (ESVS)^28^ and North American Society for Vascular Surgery (SVS)^29^ propose carotid duplex US screening for selected asymptomatic individuals with established vascular risk factors, such as smoking, hypertension, hyperlipidaemia or obesity, and diabetes mellitus. However, these recommendations require patients to actively seek medical care to participate in this screening. Given the widespread use of panoramic imaging in dentistry, there is an untapped opportunity for early cardiovascular risk assessment and the detection of individuals who might need further screening. Integrating dental observations into medical screening pathways could enhance interprofessional collaboration and improve the prevention of atherosclerotic events.

However, despite increasing evidence of the clinical relevance of detecting CCAAs on PRs, their identification by dental practitioners remains insufficient. Previous studies have reported low diagnostic sensitivity for detecting CCAA radiopaque masses on PRs,^14^^,^^30^ with many dentists confusing them with calcified anatomical structures, such as the thyroid and triticeous cartilage, hyoid bone, stylohyoid ligament, or epiglottic cartilage.^9^^,^^31^ MacDonald et al^32^ proposed a diagnostic flowchart to aid dentists in systematically identifying carotid calcifications; however, overall diagnostic knowledge in this area is still insufficient. Encouragingly, a brief training program can provide long-term skill improvement among general dentists.^33^ Furthermore, artificial intelligence has exhibited high diagnostic accuracy in identifying CCAAs on PRs, with sensitivity reaching 0.92 and a specificity of 0.96.^34^

Current guidelines do not recommend population-wide screening for asymptomatic carotid artery disease, even with non-invasive and non-ionizing US imaging. Considering the results of this study, incidental findings of bilateral CCAAs on PRs taken on odontological indication may serve as a valuable risk indicator and warrant further assessment with US or assessment of cardiovascular risk factors. This is further supported by the fact that atherosclerotic plaques in the carotid arteries seem more vulnerable, causing more thrombosis than plaques in the femoral arteries, strengthening the importance of identifying CCAAs at early stages.^35^

## Conclusion

Bilateral CCAAs were a common finding on PRs and an indicator of an increased risk of future cardiovascular events and early death among both controls and patients in the PAROKRANK study. This indicates that detection of CCAAs on PRs within both general and specialized dentistry can contribute to identifying individuals in need of medical attention and prevention of cardiovascular events and early death.
